# Supplementary material for: A new gene set identifies senescent cells and predicts senescence-associated pathways across tissues
Source: Nat Commun. 2022 Aug 16;13:4827. doi: 10.1038/s41467-022-32552-1 (PMC9381717; doi:10.1038/s41467-022-32552-1)
Supplement: Supplementary file 1 — Supplementary Information [file 41467_2022_32552_MOESM1_ESM.pdf]

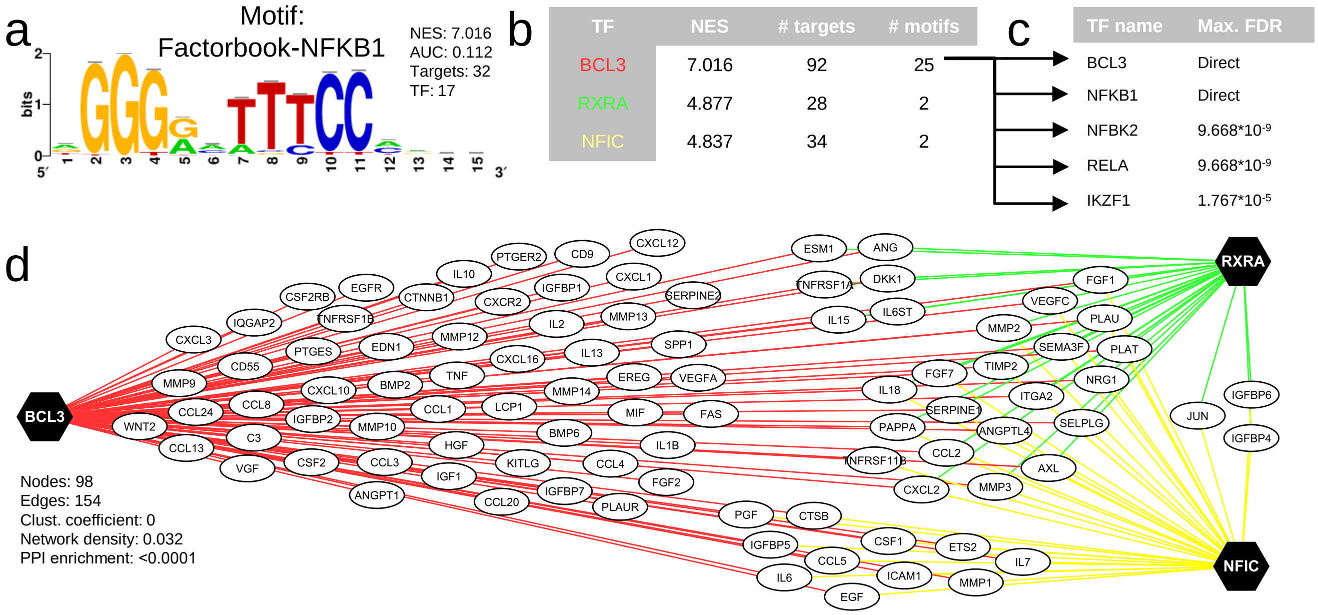

Supplementary Figure 1. *iRegulon (Janky et al. 2014) predicts the key regulons for the SASP cells.* (a) The motif with the highest enrichment for the SASP genes, according to iRegulon, was Factorbook-NFKB1. (b) The most important transcription factors with 92, 28 and 34 targets within SenMayo were BCL3, RXRA and NFIC, respectively. The first transcription factor controlled (c) BCL3, NFKB1 and -2, RELA and IKZF1, thus confirming the RNA-Seq predictions in the young and old dataset (Fig. 1B). (d) The three transcription factors control a majority (95/125) of SenMayo genes. Source data are provided as a Source Data file.

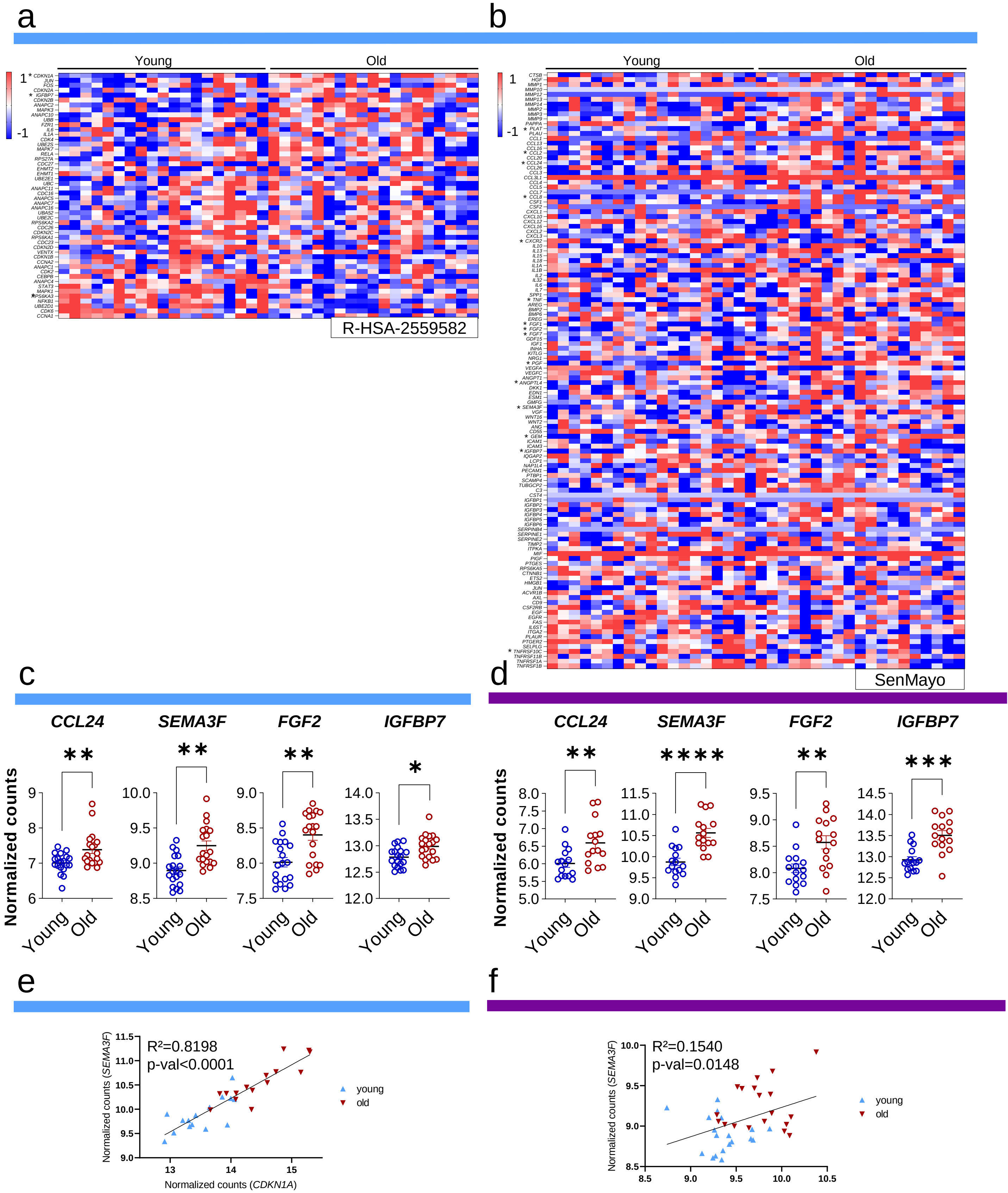

Supplementary Figure 2. *The SenMayo gene set predicts aging in two mRNA-seq data sets.* Out of the 50 available genes in the R-HSA-2559582 gene set, two were significantly enriched in the aging cohort (a), while 13 out of 125 of the SenMayo genes were enriched (b). Canonical markers of the SASP such as *CCL24, SEMA3F, FGF2, and IGFBP7* were upregulated with aging in the RNA-seq of human bone/bone marrow samples in (c) cohort A ((CCL24: and IGFBP7 two-sided unpaired t-test, SEMA3F and FGF2: Kolmogorov-Smirnov test, *CCL24*: p=0.062, *SEMA3F*: p=0.001, *FGF2:* p=0.0034, *IGFBP7*: p=0.0022), and (d) cohort B (two-sided unpaired t-test, *CCL24*: p=0.0060, *SEMA3F*: p<0.0001, *FGF2*: p=0.0028, *IGFBP7*: p=0.0001). Moreover, the senescence markers, *CDKN1A/*$p21^{CIP1}$ and SEMA3F, correlate with each other in cohort A (spearman-correlation, p<0.0001, e) and cohort B (spearman-correlation, p=0.0148, f), demonstrating a potential circumvention of high interindividual variability by combining more than one gene. *p<0.05, **p<0.01, ***p<0.001, ****p<0.0001. Cohort A (blue): n=38 (19 young, 19 old, all female), Cohort B (purple): n=30 (15 young, 15 old, all female). Depicted are mean ± SEM. Source data are provided as a Source Data file.

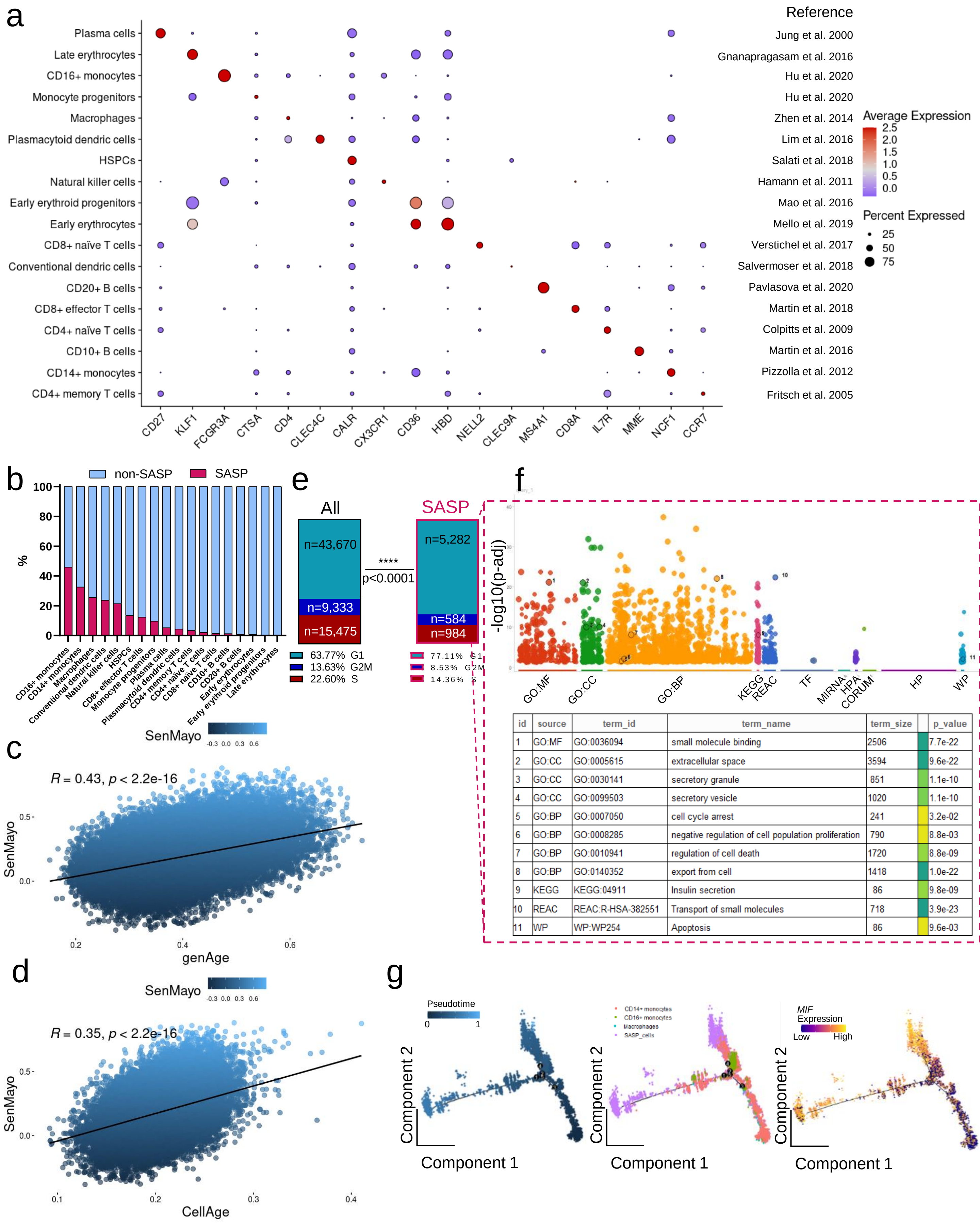

id	source	term_id	term_name	term_size	p_value
1	GO:MF	GO:0036094	small molecule binding	2506	7.7e-22
2	GO:CC	GO:0005615	extracellular space	3594	9.6e-22
3	GO:CC	GO:0030141	secretory granule	851	1.1e-10
4	GO:CC	GO:0099503	secretory vesicle	1020	1.1e-10
5	GO:BP	GO:0007050	cell cycle arrest	241	3.2e-02
6	GO:BP	GO:0008285	negative regulation of cell population proliferation	790	8.8e-03
7	GO:BP	GO:0010941	regulation of cell death	1720	8.8e-09
8	GO:BP	GO:0140352	export from cell	1418	1.0e-22
9	KEGG	KEGG:04911	Insulin secretion	86	9.8e-09
10	REAC	REAC:R-HSA-382551	Transport of small molecules	718	3.9e-23
11	WP	WP:WP254	Apoptosis	86	9.6e-03

Supplementary Figure 3. *The senescent phenotype of SASP cells in human hematopoietic bone marrow.* (a) Dotplot depicting the canonical marker genes per cluster along with the reference for each marker and cell type. (b) 46% of CD16^{+} monocytic cells had (high) SASP expression pattern; (spearman-correlation, p<0.0001, c) Bivariate correlation plots with Spearman correlation for genAge (R=0.43) and (spearman-correlation, p<0.0001, d) CellAge (R=0.35) show reliable correlations with SenMayo; e) The SASP cell cluster displayed a shift towards the G1 cell cycle phase, suggesting reduced replicative potential (Chi-square test, p<0.0001), f) The enriched terms of the SASP cluster, depicted in a Manhattan plot, show the high expression of cell cycle arrest (GO: 0007050), apoptosis (WP: WP254), and negative proliferation patterns (GO: 0008285) (multiple t-test with Benjamini-Hochberg adjustment); (g) SASP cells emerged in the final phases of cellular differentiation and increased *MIF* expression (yellow on the left, late phase) at their late developmental phase as revealed by pseudotime analysis. ****p<0.0001, n= 22 (10 male, 12 female). Source data are provided as a

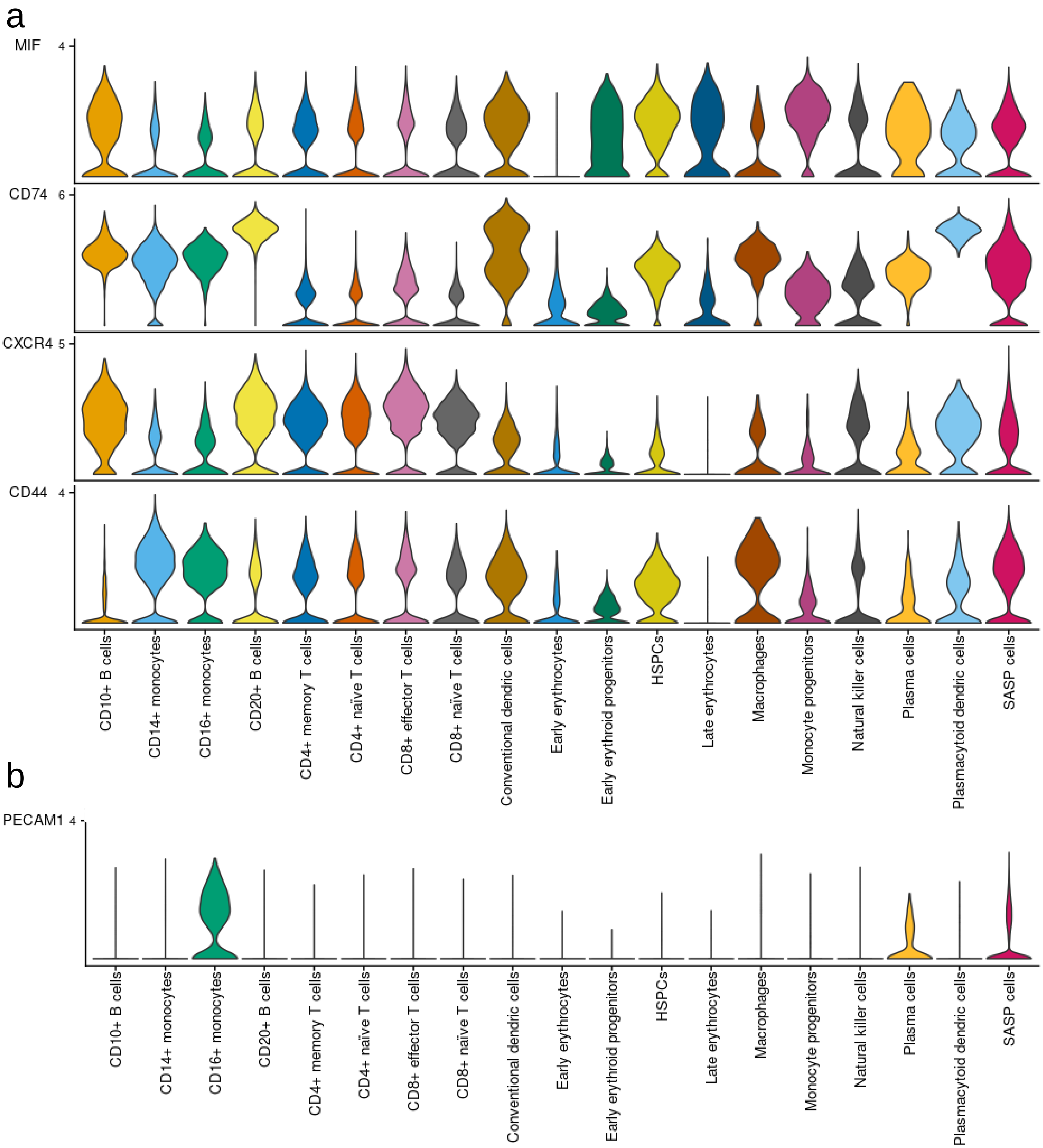

Supplementary Figure 4. *MIF and PECAM pathways in human hematopoietic bone marrow cell types.* (a) The MIF pathway and its key members show a highly heterogeneous expression pattern among all cell clusters. While $CD10^+$ B cells show a high expression of *MIF*, *CD74* and *CXCR4*, the expression of *CD44* is low. An overall high expression of all MIF members is evident in $CD8^+$ effector T cells and conventional dendritic cells and SASP cells. (b) The PECAM pathway shows an expression of PECAM1 in $CD16^+$ monocytes, plasma cells and SASP cells. Source data are provided as a Source Data file.

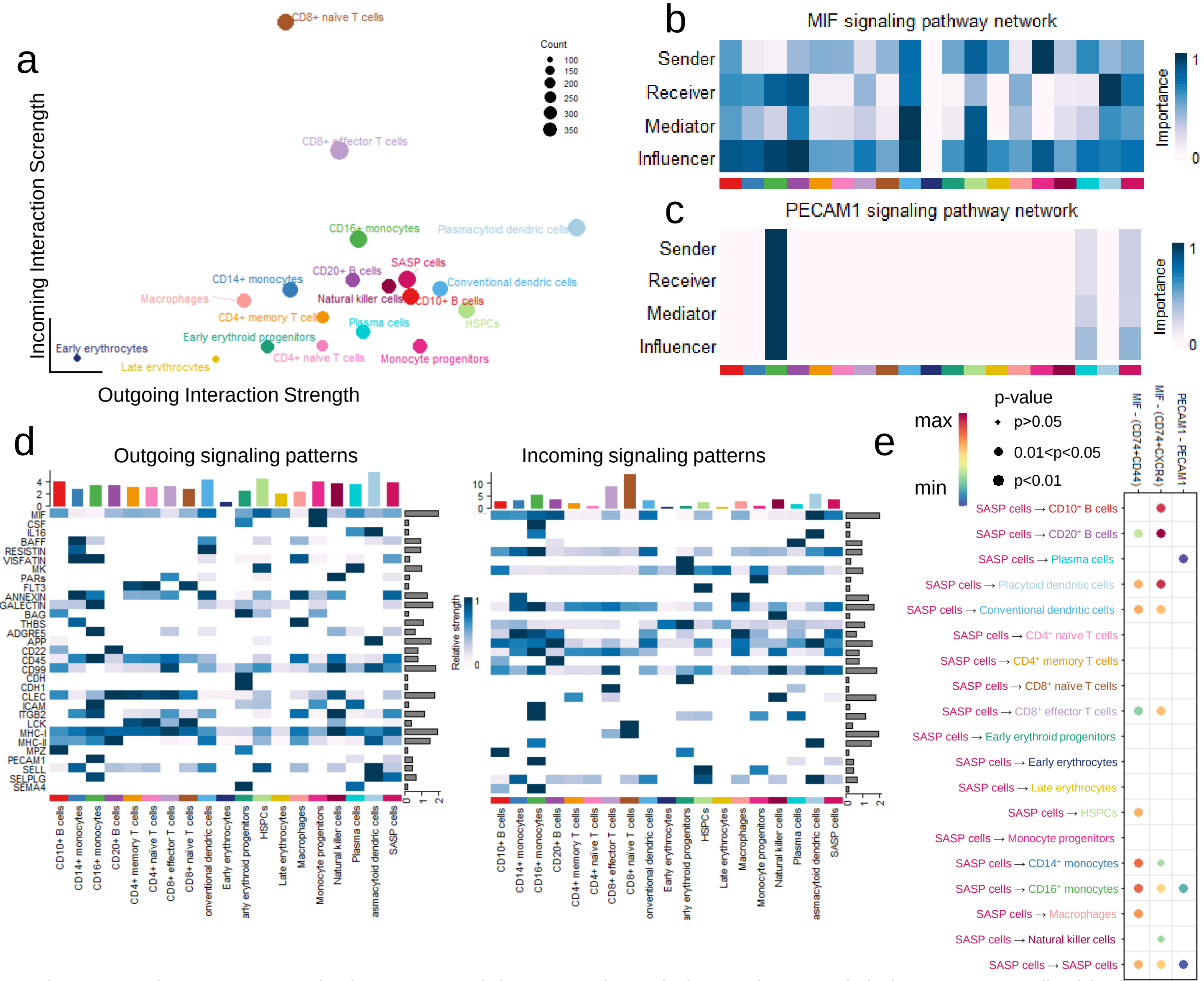

Supplementary Figure 5. *Communication patterns of the SASP cluster in human hematopoietic bone marrow cells.* (a) The SASP cluster has an overall high outgoing and moderate incoming interaction strength; (b) The SASP cells exerted various signaling functions (sender, receiver, mediator, and influencer) in the MIF pathway and (c) the PECAM1 pathway, which was used mostly by CD16$^+$ monocytes, plasma cells, and SASP cells (color-code in D); (d) The outgoing signaling pattern revealed the relevance of the MIF pathway among all other pathways, while the relative incoming signaling pattern was likewise substantial; (e) A direct MIF-driven interaction (*via* CD74/CD44 or CD74/CXCR4) from the SASP cells was detected among the majority of other cell types, especially plasmacytoid dendritic cells and B cells, while the PECAM1 pathway mostly targeted the abovementioned three cell types. *p*-values computed from one-sided permutation test, n= 22 (10 male, 12 female). Source data are provided as a Source Data file.

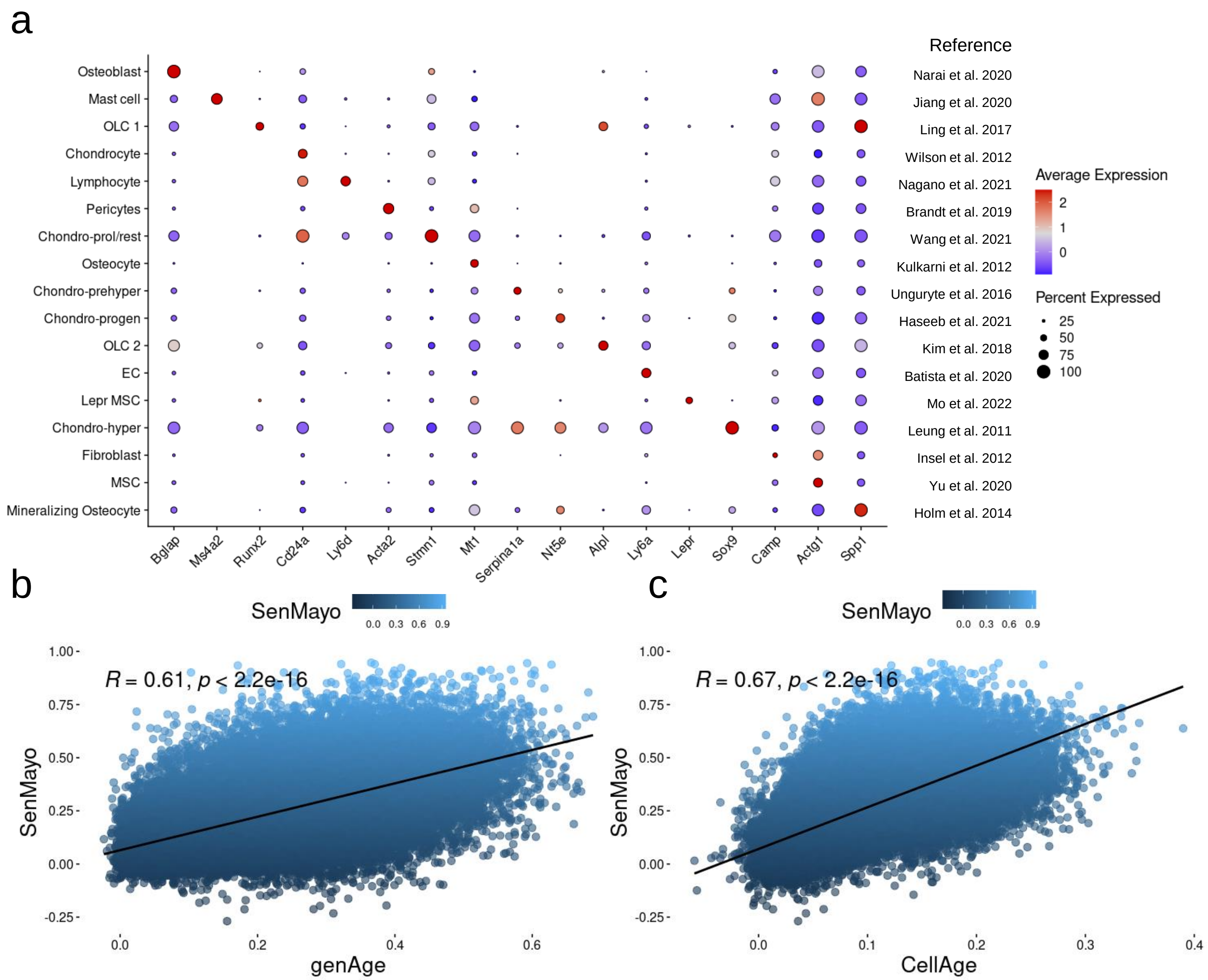

Supplementary Figure 6. *Key marker genes and correlation plots for genAge and CellAge in the murine dataset.* (a) A dotplot indicating cell cluster marker genes along with the references for each cell type. (b) A bivariate correlation plots with Spearman correlation indicate the reliable correlation of SenMayo with genAge (Spearman-correlation, p<0.0001, R=0.61) and (c) CellAge (Spearman-correlation, p<0.0001, R=0.67). Source data are provided as a Source Data file.

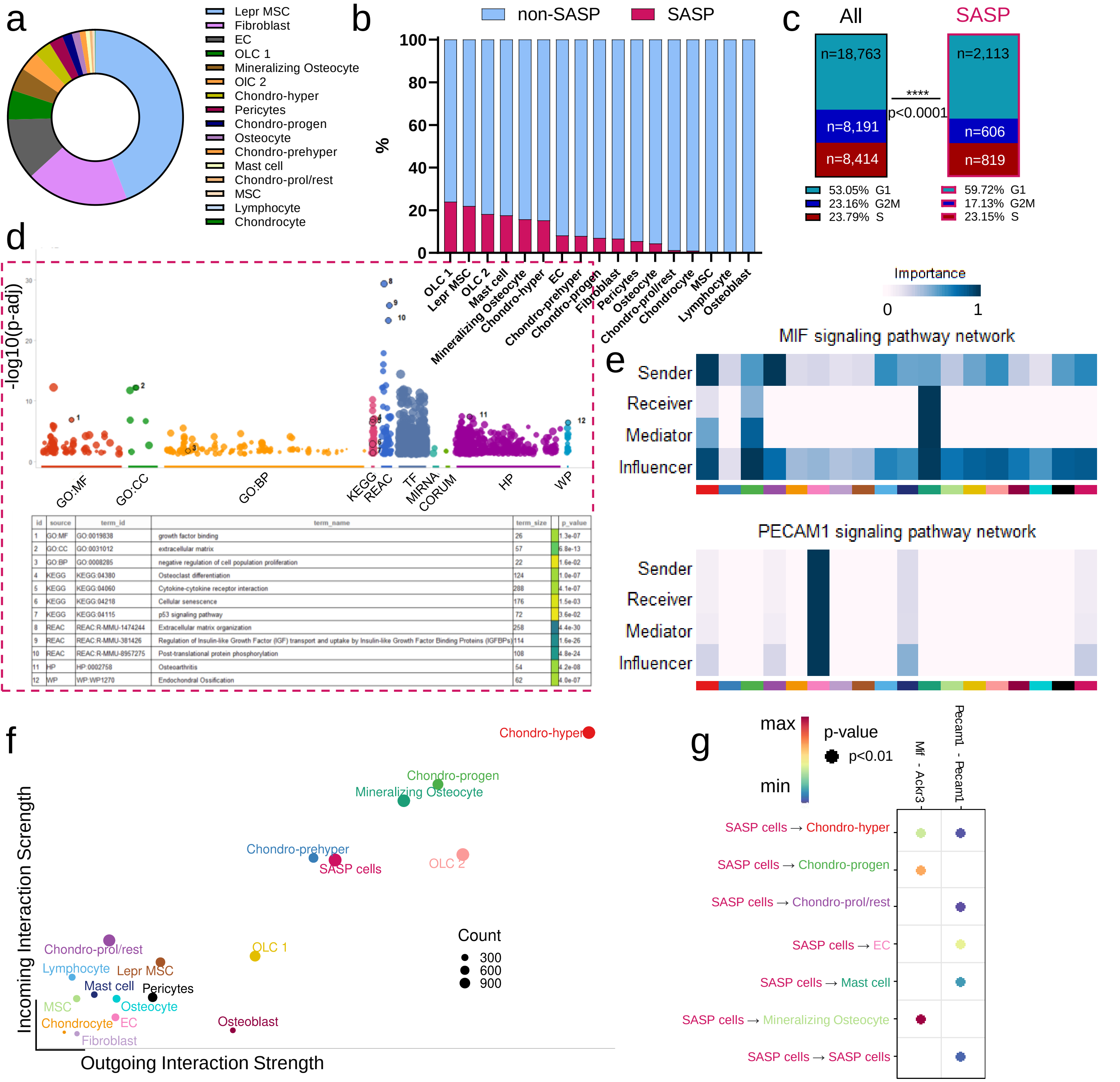

id	source	term_id	term_name	term_size		p_value
1	GO:MF	GO:0019838	growth factor binding	26		1.3e-07
2	GO:CC	GO:0031012	extracellular matrix	57		6.8e-13
3	GO:BP	GO:0008285	negative regulation of cell population proliferation	22		1.6e-02
4	KEGG	KEGG:04380	Osteoclast differentiation	124		1.0e-07
5	KEGG	KEGG:04060	Cytokine-cytokine receptor interaction	288		4.1e-07
6	KEGG	KEGG:04218	Cellular senescence	176		1.5e-03
7	KEGG	KEGG:04115	p53 signaling pathway	72		3.6e-02
8	REAC	REAC:R-MMU-1474244	Extracellular matrix organization	258		4.4e-30
9	REAC	REAC:R-MMU-381426	Regulation of Insulin-like Growth Factor (IGF) transport and uptake by Insulin-like Growth Factor Binding Proteins (IGFBPs)	114		1.6e-26
10	REAC	REAC:R-MMU-8957275	Post-translational protein phosphorylation	108		4.8e-24
11	HP	HP:0002758	Osteoarthritis	54		4.2e-08
12	WP	WP:WP1270	Endochondral Ossification	62		4.0e-07

Supplementary Figure 7. *Murine SASP cells in mesenchymal cells from bone and bone marrow are mainly of osteolineage origin and communicate via MIF.* (a) SASP cells were mostly recruited from osteolineage cells (OLC), and leptin-positive (Lep^+) mesenchymal stem cells (MSCs), while (b) 24% of OLC 1 and 18% of OLC2 cells were SASP cell members; (c) Mesenchymal SASP cells in murine bone and bone marrow significantly changed their replicative state from G2M to G1, indicating a replicative stop (Chi-square test, p<0.0001); (d) A Manhattan plot depicts an enrichment of genes associated with cellular senescence (KEGG 04218), negative regulation of proliferation (GO0008285), and cytokine-receptor interaction (KEGG 04060) within the SASP cluster (multiple t-test with Benjamini-Hochberg adjustment). (e) SASP cells function as both senders and influencers within the *MIF* network, and mostly as influencers in the PECAM1 network; (f) The outgoing interaction strength of the SASP cells was high, while they simultaneously showed a substantial incoming signaling strength; (g) Direct cell-cell interactions in the *MIF* pathway from the SASP cells is predominantly directed to hypertrophic chondrocytes, chondrocytic progenitors, and mineralizing osteocytes, while the Pecam1 pathway is directed to chondrocytes, endothelial cells, mast cells, and the SASP cells themselves. *p*-values computed from one-sided permutation test. ****p<0.0001, n= 8 (4 bone, 4 bone marrow, all male). Source data are provided as a Source Data file.

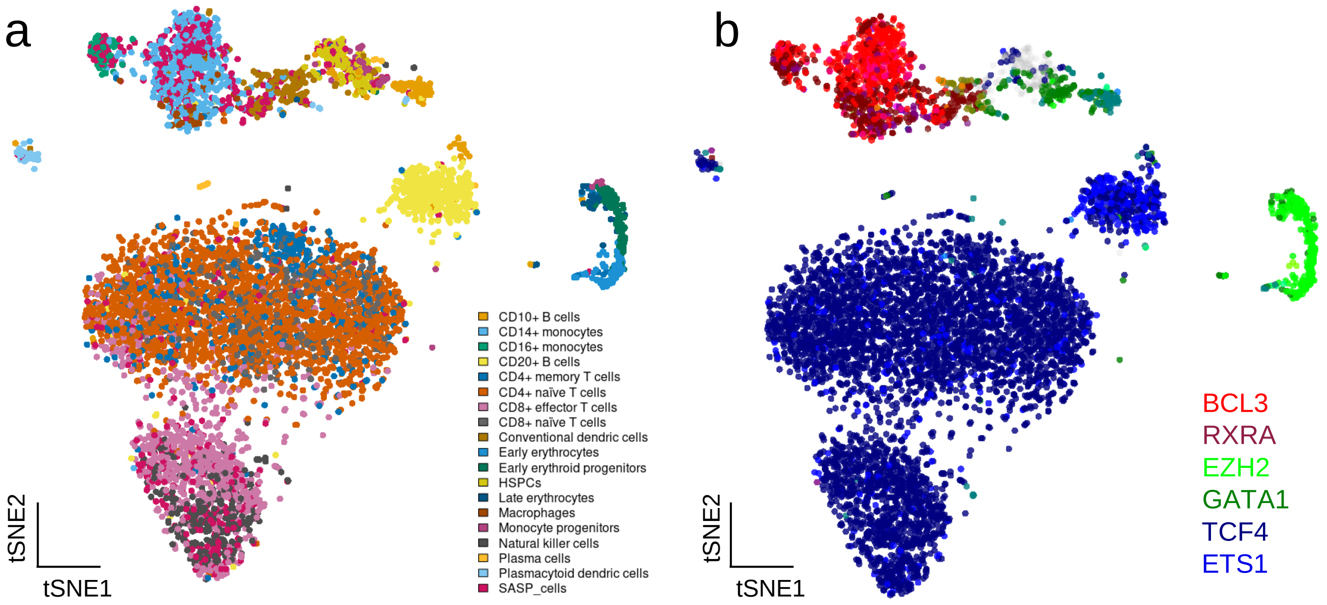

Supplementary Figure 8. *SCENIC (Aibar et al. 2017) predicts the key regulons for the SASP cells within the human single cell dataset.* (a) A regulon-based tSNE is constructed, where the SASP cells contribute substantially to the upper-middle continent (purple). (b) The predicted regulons, BCL3 and RXRA, indeed control the SASP cells containing continent. Source data are provided as a Source Data file.

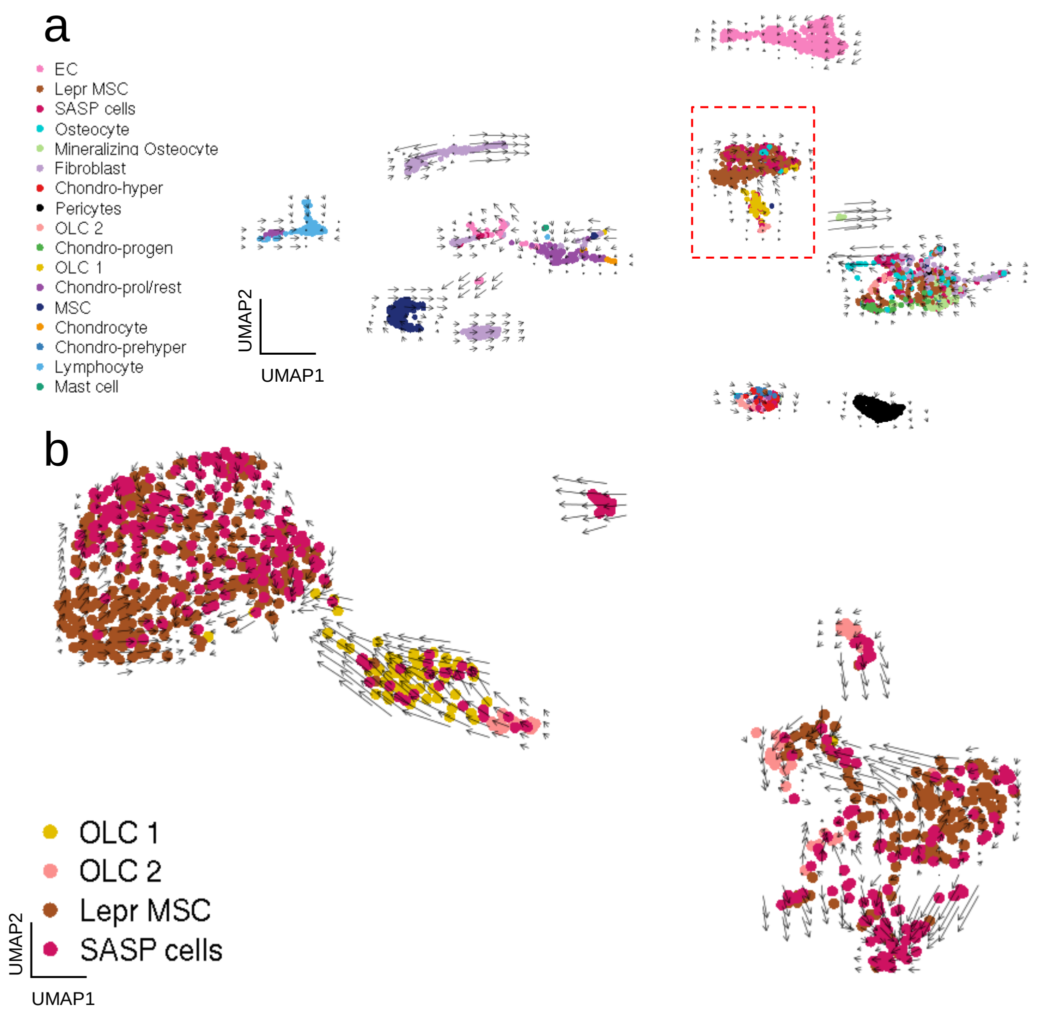

Supplementary Figure 9. *Trajectory interference using velocity (La Manno et al. 2018).* (a) The overall trajectory interference shows that SASP cells are mostly developing from OLC1, OLC2 and Lepr$^+$ MSCs. (b) A focus on these four cell types reveals the OLC1 and Lepr$^+$ MSCs as main origin of the upper-left continent, and the OLC2 and Lepr$^+$ MSCs as the origin of a different SASP cell population in the bottom-right continent. Source data are provided as a Source Data file.

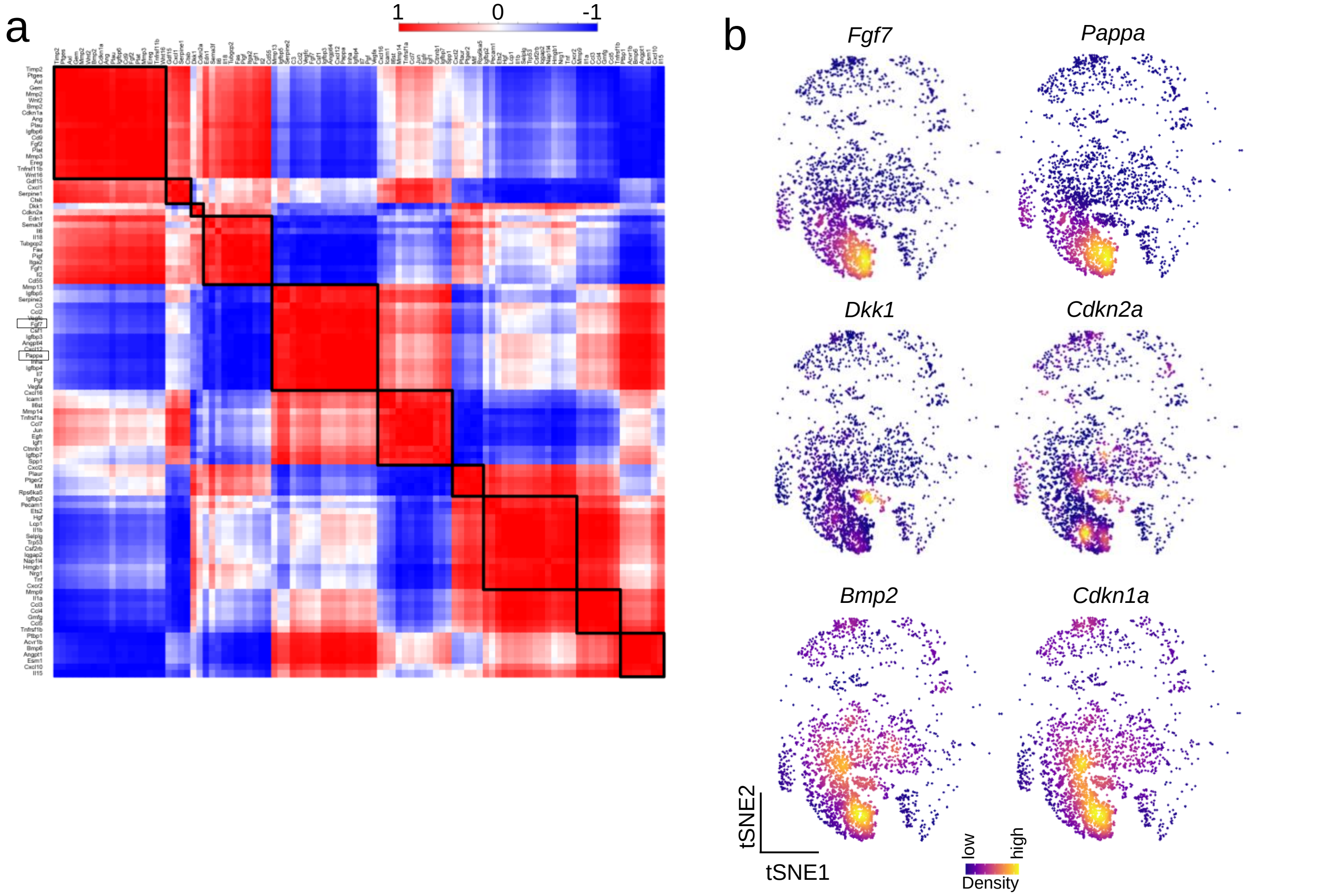

Supplementary Figure 10. *The murine SASP cluster showed an enrichment of senescent pathways and contained distinct expression modules.* (a) Within the SASP cluster, different expression modules were mathematically predicted. In this module, *Pappa* and *Fgf7* are present, which can be visualized spatially in (b) tSNE, having a similar kernel-weighed density. Other predicted co-expressional patterns were demonstrated by the pairs *Dkk1-Cdkn2a* and *Bmp2-Cdkn1a*. n= 8 (4 bone, 4 bone marrow, all male). Source data are provided as a Source Data file.

Supplementary References

1. Jung, J., Choe, J., Li, L. & Choi, Y. S. Regulation of CD27 expression in the course of germinal center B cell differentiation: the pivotal role of IL-10. *European journal of immunology* **30,** 2437–2443; 10.1002/1521-4141(2000)30:8<2437::AID-IMMU2437>3.0.CO;2-M (2000).
2. Gnanapragasam, M. N. *et al.* EKLF/KLF1-regulated cell cycle exit is essential for erythroblast enucleation. *Blood* **128,** 1631–1641; 10.1182/blood-2016-03-706671 (2016).
3. Hu, Y. *et al.* Genetic landscape and autoimmunity of monocytes in developing Vogt-Koyanagi-Harada disease. *Proceedings of the National Academy of Sciences of the United States of America* **117,** 25712–25721; 10.1073/pnas.2002476117 (2020).
4. Zhen, A. *et al.* CD4 ligation on human blood monocytes triggers macrophage differentiation and enhances HIV infection. *Journal of virology* **88,** 9934–9946; 10.1128/JVI.00616-14 (2014).
5. Lim, S. M. *et al.* CLEC4C p.K210del variant causes impaired cell surface transport in plasmacytoid dendritic cells of amyotrophic lateral sclerosis. *Oncotarget* **7,** 24942–24949; 10.18632/oncotarget.7886 (2016).
6. Salati, S. *et al.* Calreticulin Affects Hematopoietic Stem/Progenitor Cell Fate by Impacting Erythroid and Megakaryocytic Differentiation. *Stem cells and development* **27,** 225–236; 10.1089/scd.2017.0137 (2018).
7. Hamann, I. *et al.* Analyses of phenotypic and functional characteristics of CX3CR1-expressing natural killer cells. *Immunology* **133,** 62–73; 10.1111/j.1365-2567.2011.03409.x (2011).
8. Mao, B. *et al.* Early Development of Definitive Erythroblasts from Human Pluripotent Stem Cells Defined by Expression of Glycophorin A/CD235a, CD34, and CD36. *Stem cell reports* **7,** 869–883; 10.1016/j.stemcr.2016.09.002 (2016).
9. Mello, F. V. *et al.* Maturation-associated gene expression profiles during normal human bone marrow erythropoiesis. *Cell death discovery* **5,** 69; 10.1038/s41420-019-0151-0 (2019).
10. Verstichel, G. *et al.* The checkpoint for agonist selection precedes conventional selection in human thymus. *Science immunology* **2**; 10.1126/sciimmunol.aah4232 (2017).
11. Salvermoser, J. *et al.* Clec9a-Mediated Ablation of Conventional Dendritic Cells Suggests a Lymphoid Path to Generating Dendritic Cells In Vivo. *Frontiers in immunology* **9,** 699; 10.3389/fimmu.2018.00699 (2018).
12. Pavlasova, G. & Mraz, M. The regulation and function of CD20: an "enigma" of B-cell biology and targeted therapy. *Haematologica* **105,** 1494–1506; 10.3324/haematol.2019.243543 (2020).
13. Martin, M. D. & Badovinac, V. P. Defining Memory CD8 T Cell. *Frontiers in immunology* **9,** 2692; 10.3389/fimmu.2018.02692 (2018).
14. Colpitts, S. L., Dalton, N. M. & Scott, P. IL-7 receptor expression provides the potential for long-term survival of both CD62Lhigh central memory T cells and Th1 effector cells during Leishmania major infection. *Journal of immunology (Baltimore, Md. : 1950)* **182,** 5702–5711; 10.4049/jimmunol.0803450 (2009).
15. Martin, V. G. *et al.* Transitional B Cells in Early Human B Cell Development - Time to Revisit the Paradigm? *Frontiers in immunology* **7,** 546; 10.3389/fimmu.2016.00546 (2016).
16. Pizzolla, A. *et al.* Reactive oxygen species produced by the NADPH oxidase 2 complex in monocytes protect mice from bacterial infections. *Journal of immunology (Baltimore, Md. : 1950)* **188,** 5003–5011; 10.4049/jimmunol.1103430 (2012).
17. Fritsch, R. D. *et al.* Stepwise differentiation of CD4 memory T cells defined by expression of CCR7 and CD27. *Journal of immunology (Baltimore, Md. : 1950)* **175,** 6489–6497; 10.4049/jimmunol.175.10.6489 (2005).
18. Janky, R. *et al.* iRegulon: from a gene list to a gene regulatory network using large motif and track collections. *PLoS computational biology* **10,** e1003731; 10.1371/journal.pcbi.1003731 (2014).
19. Aibar, S. *et al.* SCENIC: single-cell regulatory network inference and clustering. *Nature methods* **14,** 1083–1086; 10.1038/nmeth.4463 (2017).
20. La Manno, G. *et al.* RNA velocity of single cells. *Nature* **560,** 494–498; 10.1038/s41586-018-0414-6 (2018).
21. Narai, T. *et al.* Establishment of human immortalized mesenchymal stem cells lines for the monitoring and analysis of osteogenic differentiation in living cells. *Heliyon* **6,** e05398; 10.1016/j.heliyon.2020.e05398 (2020).
22. Jiang, J. *et al.* Gene signatures from scRNA-seq accurately quantify mast cells in biopsies in asthma. *Clinical and experimental allergy : journal of the British Society for Allergy and Clinical Immunology* **50,** 1428–1431; 10.1111/cea.13732 (2020).
23. Wilson, R. *et al.* Changes in the chondrocyte and extracellular matrix proteome during post-natal mouse cartilage development. *Molecular & cellular proteomics : MCP* **11,** M111.014159; 10.1074/mcp.M111.014159 (2012).
24. Nagano, T. *et al.* LY6D-induced macropinocytosis as a survival mechanism of senescent cells. *The Journal of biological chemistry* **296,** 100049; 10.1074/jbc.RA120.013500 (2021).
25. Brandt, M. M. *et al.* Transcriptome analysis reveals microvascular endothelial cell-dependent pericyte differentiation. *Scientific reports* **9,** 15586; 10.1038/s41598-019-51838-x (2019).
26. Wang, X. *et al.* Comparison of the major cell populations among osteoarthritis, Kashin-Beck disease and healthy chondrocytes by single-cell RNA-seq analysis. *Cell death & disease* **12,** 551; 10.1038/s41419-021-03832-3 (2021).
27. Kulkarni, R. N. *et al.* MT1-MMP modulates the mechanosensitivity of osteocytes. *Biochemical and biophysical research communications* **417,** 824–829; 10.1016/j.bbrc.2011.12.045 (2012).
28. Unguryte, A. *et al.* Human articular chondrocytes with higher aldehyde dehydrogenase activity have stronger expression of COL2A1 and SOX9. *Osteoarthritis and cartilage* **24,** 873–882; 10.1016/j.joca.2015.11.019 (2016).
29. Haseeb, A. *et al.* SOX9 keeps growth plates and articular cartilage healthy by inhibiting chondrocyte dedifferentiation/osteoblastic redifferentiation. *Proceedings of the National Academy of Sciences of the United States of America* **118**; 10.1073/pnas.2019152118 (2021).
30. Kim, J. H., Kim, K., Kim, I., Seong, S. & Kim, N. c-Src-Dependent and -Independent Functions of Matk in Osteoclasts and Osteoblasts. *Journal of immunology (Baltimore, Md. : 1950)* **200,** 2455–2463; 10.4049/jimmunol.1700582 (2018).
31. Batista, A. R. *et al.* Ly6a Differential Expression in Blood-Brain Barrier Is Responsible for Strain Specific Central Nervous System Transduction Profile of AAV-PHP.B. *Human gene therapy* **31,** 90–102; 10.1089/hum.2019.186 (2020).
32. Mo, C. *et al.* Single-cell transcriptomics of LepR-positive skeletal cells reveals heterogeneous stress-dependent stem and progenitor pools. *The EMBO journal* **41,** e108415; 10.15252/embj.2021108415 (2022).
33. Ling, M. *et al.* Epigenetic regulation of Runx2 transcription and osteoblast differentiation by nicotinamide phosphoribosyltransferase. *Cell & bioscience* **7,** 27; 10.1186/s13578-017-0154-6 (2017).
34. Leung, V. Y. L. *et al.* SOX9 governs differentiation stage-specific gene expression in growth plate chondrocytes via direct concomitant transactivation and repression. *PLoS genetics* **7,** e1002356; 10.1371/journal.pgen.1002356 (2011).
35. Insel, P. A. *et al.* cAMP and Epac in the regulation of tissue fibrosis. *British journal of pharmacology* **166,** 447–456; 10.1111/j.1476-5381.2012.01847.x (2012).
36. Yu, X. *et al.* RNA-seq reveals tight junction-relevant erythropoietic fate induced by OCT4 in human hair follicle mesenchymal stem cells. *Stem cell research & therapy* **11,** 454; 10.1186/s13287-020-01976-1 (2020).
37. Holm, E. *et al.* Osteopontin mediates mineralization and not osteogenic cell development in vitro. *The Biochemical journal* **464,** 355–364; 10.1042/BJ20140702 (2014).
