# Supplementary material for: A new gene set identifies senescent cells and predicts senescence-associated pathways across tissues
Source: Nat Commun. 2022 Aug 16;13:4827. doi: 10.1038/s41467-022-32552-1 (PMC9381717; doi:10.1038/s41467-022-32552-1)
Supplement: Supplementary file 3 — Description of Additional Supplementary Files [file 41467_2022_32552_MOESM3_ESM.docx]

Supplementary data 1. Genes included in the SenMayo panel. The relationship of each gene to senescence/aging is described in the reference indicated. Tab 1: Human genes, Tab 2: Mouse genes.

Supplementary data 2: Network characteristics.
