# Supplementary material for: A new gene set identifies senescent cells and predicts senescence-associated pathways across tissues
Source: Nat Commun. 2022 Aug 16;13:4827. doi: 10.1038/s41467-022-32552-1 (PMC9381717; doi:10.1038/s41467-022-32552-1)
Supplement: Supplementary file 7 — Reporting Summary [file 41467_2022_32552_MOESM7_ESM.pdf]

nature portfolio

Corresponding author(s): Dominik Saul, M.D., Joshua N. Farr, Ph.D., Sundeep Khosla, M.D.

Last updated by author(s): Jun 6, 2022

Reporting Summary

Nature Portfolio wishes to improve the reproducibility of the work that we publish. This form provides structure for consistency and transparency in reporting. For further information on Nature Portfolio policies, see our Editorial Policies and the Editorial Policy Checklist.

Statistics

For all statistical analyses, confirm that the following items are present in the figure legend, table legend, main text, or Methods section.

n/a	Confirmed	
☐	☒	The exact sample size (*n*) for each experimental group/condition, given as a discrete number and unit of measurement
☐	☒	A statement on whether measurements were taken from distinct samples or whether the same sample was measured repeatedly
☐	☒	The statistical test(s) used AND whether they are one- or two-sided<br>*Only common tests should be described solely by name; describe more complex techniques in the Methods section.*
☐	☒	A description of all covariates tested
☐	☒	A description of any assumptions or corrections, such as tests of normality and adjustment for multiple comparisons
☐	☒	A full description of the statistical parameters including central tendency (e.g. means) or other basic estimates (e.g. regression coefficient) AND variation (e.g. standard deviation) or associated estimates of uncertainty (e.g. confidence intervals)
☐	☒	For null hypothesis testing, the test statistic (e.g. *F*, *t*, *r*) with confidence intervals, effect sizes, degrees of freedom and *P* value noted<br>*Give P values as exact values whenever suitable.*
☐	☒	For Bayesian analysis, information on the choice of priors and Markov chain Monte Carlo settings
☒	☐	For hierarchical and complex designs, identification of the appropriate level for tests and full reporting of outcomes
☒	☐	Estimates of effect sizes (e.g. Cohen's *d*, Pearson's *r*), indicating how they were calculated

Our web collection on statistics for biologists contains articles on many of the points above.

Software and code

Policy information about availability of computer code

Data collection	R-packages were Nebulosa (3.1394), Monocle (2.18.095), dittoSeq (1.2.696), Escape (1.0.1, "Borcherding N, Andrews J (2021). escape: Easy single cell analysis platform for enrichment. R package version 1.2.0.", Cellchat (1.4.0), bigSCale (2.170), gprofiler2 (0.2.097), PCAtools (2.4.0, Blighe K, Lun A (2021). PCAtools: PCAtools. Everything Principal Components Analysis. R package version 2.4.0), and corrplot (0.89) and SCENIC (1.2.498) and velocyto.R (0.6).
Data analysis	R4.0.2

For manuscripts utilizing custom algorithms or software that are central to the research but not yet described in published literature, software must be made available to editors and reviewers. We strongly encourage code deposition in a community repository (e.g. GitHub). See the Nature Portfolio guidelines for submitting code & software for further information.

Data

Policy information about availability of data

All manuscripts must include a data availability statement. This statement should provide the following information, where applicable:

- Accession codes, unique identifiers, or web links for publicly available datasets
- A description of any restrictions on data availability
- For clinical datasets or third party data, please ensure that the statement adheres to our policy

The RNA-seq data from Fig. 1 have been deposited in the National Center for Biotechnology Information's Gene Expression Omnibus under GSE72815 and GSE141595. The RNA-sequencing data from Mus musculus brain microglia (Fig. 2a) is deposited under (GSE145265), prefrontal cortex (Fig. 2b) under GSE128770,

dorsal hippocampus (Fig. 2c) under GSE94832 and the tabula muris senis (Fig. 2d) under GSE149590).
The human single cell sequencing data (Fig. 4) is stored at GSE120221, while the murine single cell sequencing data (Fig. 5) is stored at GSE128423.The murine INK-ATTAC tibia diaphysis bulk RNA-sequencing data (Fig. 3a-b) is available from dryad (https://datadryad.org/stash/share/YdD6C2ZFDgSizXehPR0qqPy4io7oRQJMGRlrPuij9WU) or GSE199493.The human subcutaneous fat bulk RNA-sequencing data from trial no. NCT02848131 (Fig. 3d-e) is available from dryad (https://datadryad.org/stash/share/YdD6C2ZFDgSizXehPR0qqPy4io7oRQJMGRlrPuij9WU) and PRJNA826433.Source data are provided with this paper.

Human research participants

Policy information about studies involving human research participants and Sex and Gender in Research.

Reporting on sex and gender	Information not provided to protect participant identity
Population characteristics	Subjects with diabetic kidney disease. Adipose tissue was collected before and 11 days after senolytic treatment (oral Dasatinib 100mg and Quercetin 1000mg). PMID: 31542391
Recruitment	The participants were enrolled by invitation (NCT02848131). Informed consent was obtained from study participants. Patients received $175 for fat+skin biopsy as remuneration and an additional $75 for the four month visit.
Ethics oversight	The Mayo Clinic Institutional Review Board approved this study, which is registered at ClinicalTrials.gov (NCT02848131)(PMC6796530).

Note that full information on the approval of the study protocol must also be provided in the manuscript.

Field-specific reporting

Please select the one below that is the best fit for your research. If you are not sure, read the appropriate sections before making your selection.

☒ Life sciences ☐ Behavioural & social sciences ☐ Ecological, evolutionary & environmental sciences

For a reference copy of the document with all sections, see nature.com/documents/nr-reporting-summary-flat.pdf

Life sciences study design

All studies must disclose on these points even when the disclosure is negative.

Sample size	The sample sizes were determined based on previously conducted and published experiments (e.g. PMID: 28825716, PMID: 34617510) in which statistically significant differences were observed among various bone parameters in response to multiple interventions in our laboratory.
Data exclusions	No data were excluded from the analyses.
Replication	The qPCR was performed in triplicates. All attempts at replication were successful.
Randomization	In the murine studies, animals were randomized to experimental groups by body weight. In the human trial, the randomization followed the protocol NCT02848131.
Blinding	Analyses were performed in a blinded fashion.

Reporting for specific materials, systems and methods

We require information from authors about some types of materials, experimental systems and methods used in many studies. Here, indicate whether each material, system or method listed is relevant to your study. If you are not sure if a list item applies to your research, read the appropriate section before selecting a response.

Materials & experimental systems

n/a	Involved in the study
☒	☐ Antibodies
☒	☐ Eukaryotic cell lines
☒	☐ Palaeontology and archaeology
☐	☒ Animals and other organisms
☐	☒ Clinical data
☒	☐ Dual use research of concern

Methods

n/a	Involved in the study
☒	☐ ChIP-seq
☒	☐ Flow cytometry
☒	☐ MRI-based neuroimaging

Animals and other research organisms

Policy information about studies involving animals; ARRIVE guidelines recommended for reporting animal research, and Sex and Gender in Research

Laboratory animals	scRNA-seq data from bone marrow cells isolated from C57BL/6 mice (n=14)54 and from C57BL/6JN mice (n=30)36 and from the tabula muris senis.<br>Fig. 6: Mice from PMID: 28825716. Young mice: n=12, Old Veh: n=13, Old AP n=16, all female.<br><br>Mice were housed in ventilated cages and maintained within a pathogen-free, accredited facility under a 12-hr light/dark cycle with constant temperature (23 °C) and access to food (diet details are specified below) and water ad libitum.
Wild animals	No wild animals were used in the study.
Reporting on sex	Sex is indicated in the figure legends.
Field-collected samples	Please state here that no field collected samples were used in the study.
Ethics oversight	Mouse strains and drug treatments. All animal protocols were approved by the Institutional Animal Care and Use Committee (IACUC), and all experiments were performed in accordance with IACUC guidelines.

Note that full information on the approval of the study protocol must also be provided in the manuscript.

Clinical data

Policy information about clinical studies

All manuscripts should comply with the ICMJEguidelines for publication of clinical research and a completedCONSORT checklist must be included with all submissions.

Clinical trial registration	NCT02848131
Study protocol	NCT02848131
Data collection	Location: Mayo Clinic Rochester, MN, USA. Start: July 2016. Currently running (estimated April 2023).
Outcomes	Primary Outcome Measures: Change in proportion of senescent cells present. Secondary Outcome Measurement: Change in proportion of senescent mesenchymal stem cells present. Change in mesenchymal stem cell function. Change in Frailty index score. Change in kidney function.
